# Exercise Capacity in Patients With Chronic Obstructive Pulmonary Disease Treated With Tele-Yoga Versus Tele-Pulmonary Rehabilitation: A Pilot Validation Study

**DOI:** 10.7759/cureus.30994

**Published:** 2022-11-01

**Authors:** Saloni Malik, Ruchi Dua, Ajay S Krishnan, Suresh Kumar, Sunil Kumar, Osama Neyaz, Ajeet S Bhadoria

**Affiliations:** 1 Pulmonary Medicine, All India Institute of Medical Sciences, Rishikesh, Rishikesh, IND; 2 Radiation Oncology, All India Institute of Medical Sciences, Rishikesh, Rishikesh, IND; 3 Yoga, Dev Sanskriti Vishwavidyalaya, Haridwar, IND; 4 Yoga, Dev Sanskriti Vishwavidhyala, Haridwar, IND; 5 Physical Medicine and Rehabilitation, All India Institute of Medical Sciences, Rishikesh, Rishikesh, IND; 6 Community and Family Medicine, All India Institute of Medical Sciences, Rishikesh, Rishikesh, IND

**Keywords:** copd: chronic obstructive pulmonary disease, depression, anxiety, quality of life, tele-pulmonary rehabilitation, tele-yoga

## Abstract

Background

In chronic obstructive pulmonary disease (COPD), pulmonary rehabilitation (PR) is an integral component of the non-pharmacological management of COPD. Yoga has proven to be beneficial in COPD, although well-designed comparative studies between the two modalities are lacking. This pilot study aims to compare these two modalities delivered as supervised tele-intervention.

Methods

The outpatient department (OPD) of a tertiary hospital recruited consenting, consecutive, inclusion-eligible COPD participants who were randomly assigned to intervention and control arms of 30 patients each. The intervention arm received a 45-minute tele-yoga therapy module (T-YT) validated by content validity ratio (CVR), computed using Lawshe's methodology and responses from 24 yoga specialists. The control arm received a 45-minute standardized tele-pulmonary rehabilitation session (T-PR). T-YT and T-PR were both managed through an online portal. Exercise capacity as measured by the six-minute walk distance (6MWD), symptom score (COPD assessment test [CAT], modified medical research council [mMRC]), forced expiratory volume in one second (FEV1%), quality of life (QoL) scores, St. George respiratory questionnaire (SGRQ), depression and anxiety scores (patient health questionnaire [PHQ-9] and generalized anxiety disorder scale [GAD-7] scores), were recorded at baseline and at the end of three months.

Results

6MWD, symptom scores, SGRQ, PHQ-9, and GAD-7 all improved significantly from baseline within each group, but there was no statistically significant difference between the groups. FEV1% did not differ significantly between or among groups. This study shows T-YT module can be a reasonable substitute for T-PR in patients with COPD.

Conclusions

T-YT is beneficial in patients of COPD in terms of exercise capacity, symptom scores, and depression and anxiety scores and can be a reasonable alternative to T-PR.

## Introduction

Chronic obstructive pulmonary disease (COPD) is a progressive respiratory disease that causes airflow obstruction and respiratory distress, which is often preventable and treatable. Smoking and environmental pollution are the main causative factors of this disease [1]. It can cost as much as $50 billion in healthcare expenses in countries like the United States, where COPD is the third leading cause of mortality [2]. Currently, inhaled bronchodilator therapy is the predominant treatment for COPD [3]. Pulmonary rehabilitation is a recognized, non-pharmacologic method for managing dyspnea and exercise intolerance in COPD [4]. Pulmonary rehabilitation is a supervised program consisting of fitness training, health education, and breathing methods designed specifically for people with chronic respiratory diseases.

Yoga, a form of psychophysical activity, is used in the treatment of various respiratory diseases. Yoga's three primary tools are the body, the breath, and the mind. According to studies, yoga can enhance breathing patterns, chest expansion, lung capacity, body posture, health-related quality of life, and respiratory symptoms [5]. However, yoga is not a part of non-pharmacological management in COPD patients according to national or international guidelines [6]. This validation pilot study aims to compare the effect of tele-yoga therapy (T-YT) on tele-pulmonary rehabilitation (T-PR), in addition to standard treatment, in COPD patients. The novelty of this study is that both interventions have been delivered at home via online platforms as tele-interventions. Objectives also include validating a 45-minute yoga module used as part of the intervention in this study.

## Materials and methods

Development of the yoga module

Scientific research publications connected to yoga and COPD were screened in PubMed, Scopus, and Google Scholar databases with crucial phrases: yoga and COPD. Based on the literature review, we found a protocol performed by an Indian study on COPD patients, including Asana, Kriya, and Pranayama. The module is referred to as the "45-minute yoga module" [7].

Validation procedure

The 45-minute yoga module's components were entered into a Google form. Each item was described and accompanied by three options: a) not necessary, b) useful but not essential, and c) extremely essential. This Google form was sent to twenty-four yoga specialists for validation and feedback on its improvement and limitations. Three years of postgraduate experience in the field of yoga was the bare minimum requirement for a yoga specialist. Responses of experts were recorded in an Excel spreadsheet and used to calculate the content validity ratio (CVR) for each item, item-level content validity index (I-CVI), scale-level content validity index based on the average method (S-CVI), scale-level content validity index based on the universal agreement method (S-CVI/UA), and Cronbach's alpha for item group reliability (0.73) [8].

Patient selection

All COPD patients who presented consecutively to the outpatient department at All India Institute of Medical Sciences (AIIMS), Rishikesh, were screened. In accordance with the Global Initiative for Chronic Obstructive Lung Disease (GOLD) guidelines, consenting and stable diagnosed cases between 40-75 years of age and belonging to group B-D were enrolled [5]. Exclusion criteria were acute congestive heart failure, uncontrolled diabetes mellitus, severe uncontrolled hypertension, chronic liver disease, chronic renal failure, significant co-morbidity limiting acute life expectancy, pregnant and lactating mothers, patients in acute exacerbation, unable to perform rehabilitation, exercises (e.g., severe visual, auditory or neurological or musculoskeletal involvement) or unable to perform spirometry. 

Study design

This two-arm pilot validation study was conducted at AIIMS, Rishikesh, between January 2021 and June 2021 and registered with the clinical trials registry of India (CTRI) with the number: CTRI/2020/11/029249. The study adhered to the standards of the Helsinki Declaration [9].

Randomization and blinding

Participants who agreed to participate and met the inclusion criteria were randomly assigned to either the intervention or the control arm. The computer was responsible for producing the sequence of randomization. Opaque, sequentially numbered, sealed envelopes were used for group allocation.

Blinding of participants was not possible, but the outcome assessor and statistician were blinded. 

Intervention arm

The participants were initially trained by a qualified yoga instructor in at least two sittings in OPD to ensure the adequate performance of the validated module at home. Participants then performed 45 minutes of supervised home T-YT sessions five days/per week for three months. In addition, usual treatment as per guidelines, counseling for adherence, the technique of inhaled medication, and nutrition were also provided. The participants visited OPD on a monthly basis or as per need for three months. Compliance was defined as >3 supervised sessions per week for three months.

Control arm

The participants were initially trained by a qualified physiotherapist in at least two sittings in OPD to ensure the adequate performance of the module at home [10]. Participants then performed 45 minutes of supervised home T-PR sessions for three months. In addition, usual treatment as per guidelines, counseling for adherence, the technique of inhaled medication, and nutrition were also provided. The participants visited OPD on a monthly basis or as per need for three months. Compliance was defined as >3 supervised sessions per week for three months.

Outcomes

The primary outcome was exercise capacity in terms of a six-minute walk distance (6MWD). Secondary outcomes included forced expiratory volume in one second (FEV1%), symptom score COPD assessment test (CAT) and modified medical research council (mMRC) dyspnea scale, health-related quality of life - St. George respiratory questionnaire (SGRQ) Hindi validated tool), patient health questionnaire (PHQ-9) Hindi tool, and generalized anxiety disorder scale (GAD-7) score, Hindi tool [11-15]. All the outcomes were recorded at baseline and at the end of the third month.

Statistical analysis

For the entirety of the module, S-CVI/UA was calculated using the universal agreement technique, and the CVR value was determined using the Lawshe method. Ayre and Scally (2014) report that the crucial value of CVR for a group of 24 experts is 0.47. Thus, items with a CVR of 0.47 or above were retained for the 45-minute yoga module [16]. The CVR results of the yoga module demonstrated that these specific practices are considered essential for COPD by medical specialists.

We used either parametric or non-parametric tests based on the outcomes of the Kolmogorov-Smirnov (KS) test. For parametric data, we utilized independent and paired t-tests. For non-parametric data, we utilized the Chi-Square test, Mann-Whitney U test, and Wilcoxon signed-rank test (Table 1).

**Table 1:** Validation of yoga protocol N_e _- total number of experts replied; N - total no of experts; CVR - content validity ratio; I-CVI - item-level content validity index; S-CVI/UA - scale-level content validity index based on the universal agreement method

Yoga practice items	N_e_	N	N/2	N-N_e_/2	CVR	Remarks	I-CVI	Sum of I-CVI	S-CVI/UA	Cronbach's alpha
Pranayama and Kriya										
Anulom Vilom (5-10 cycles in five minutes)	21	24	12	9	0.75	Retained	0.88	7.63	0.85	0.73
Bhastrika ending with Suryabhedi (20-100 strokes in five minutes)	20	24	12	8	0.67	Retained	0.84			
Kapalbhati (20-100 strokes in five minutes)	18	24	12	6	0.5	Retained	0.75			
Bhramari (5-10 cycles in five minutes)	22	24	12	10	0.84	Retained	0.92			
Asanas										
Tadasana (2-4 rounds with holding in one minute)	20	24	12	8	0.67	Retained	0.84			
Surya Namaskar (2-4 complete rounds with 24 steps each in 10 minutes)	22	24	12	10	0.84	Retained	0.92			
Sukhasana (1-2 rounds with holding in one minute)	20	24	12	8	0.67	Retained	0.84			
Paschimotanasana (2-4 rounds with holding in two minutes)	21	24	12	9	0.75	Retained	0.88			
Savasana (one round in 10 minutes)	19	24	12	7	0.59	Retained	0.80			

## Results

**Figure 1:**
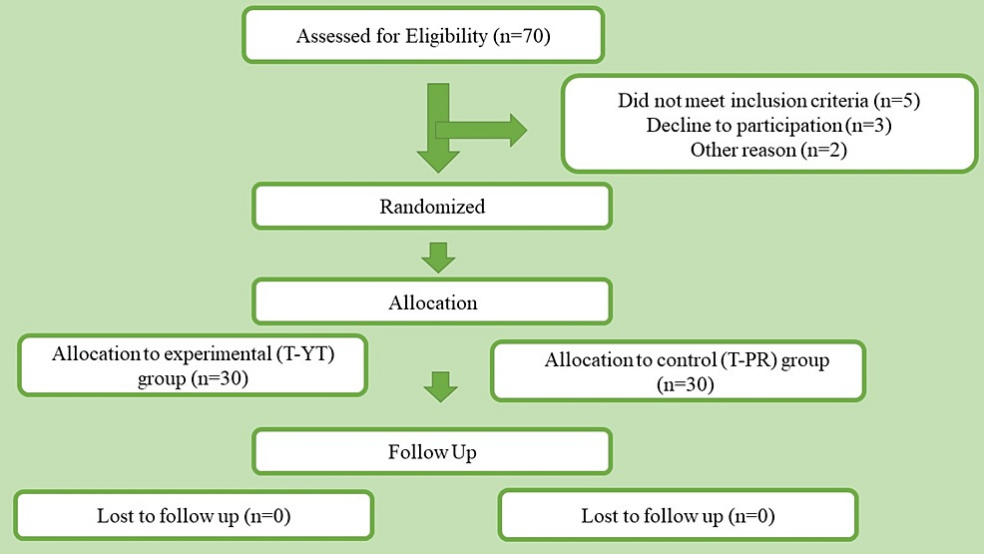
Consort chart T-YT - tele-yoga therapy; T-PR - tele-pulmonary rehabilitation

Sixty COPD participants were enrolled and randomly assigned to the intervention or control arm between January 2021 and June 2021 as per predefined inclusion/exclusion criteria (Figure 1). Baseline demographics are shown in Table 2. As observed, both groups' mean ages are 61.2±7.1 and 61.0±8.2, with 8.34% of females in both groups. At baseline, FEV1% of the T-YT group was 47.4±13.0, while for the T-PR group, it was 52.9±20.4. Both groups showed no significant difference in the age distribution (p=0.85), gender distribution (p=0.64), education (p=0.95), a combined assessment of COPD (p=0.25), 6MWD (p=0.848), FEV1% (p=0.276), CAT scores (p=0.484), mMRC score (p=0.198), SGRQ (p=0.697) and PHQ-9 (p=0.352) scores. A significant difference in smoking status (p=0.01) and GAD-7 (p=0.042) was seen at baseline between the groups. 

**Table 2:** Baseline clinical characteristics 6MWD - six-minute walk distance; CAT - COPD assessment test; mMRC- modified medical research council scale; SGRQ - St. George respiratory questionnaire; PHQ-9 - patient health questionnaire; GAD-7 - generalised anxiety disorder scale; FEV1% - forced expiratory volume in one second; COPD - chronic obstructive pulmonary disease

Characteristics	T-YT (n=30)	T-PR (n=30)
Age mean±SD (years)	61.2±7.1	61.0±8.2
Gender		
Men (%)	93.40%	90%
Women (%)	6.60%	10%
Education (%)		
Middle school	63.3	66.7
High school	26.7	20
Higher secondary	3.3	6.7
Graduation	3.3	3.3
Postgraduation	3.3	3.3
Combined assessment of COPD (%)		
B	36.67	46.67
C	20	30
D	43.34	23.34
Smoking (%)		
Non-smoker	0	23.34
Ex-smoker	76.67	56.67
Current smoker	23.34	20
Exercise capacity (6MWD)	240 (120-510)	240 (120-660)
CAT mean±SD	13.7±4.7	14.6±4.8
mMRC	3 (1-4)	3 (2-4)
SGRQ mean±SD	50.35±13.70	48.91±14.84
PHQ-9 mean±SD	10.6±3.6	9.7±3.9
GAD-7 mean±SD	8.7±3.7	6.8±3.6
FEV1% mean±SD	47.4±13.0	52.9±20.4

Exercise capacity

**Figure 2:**
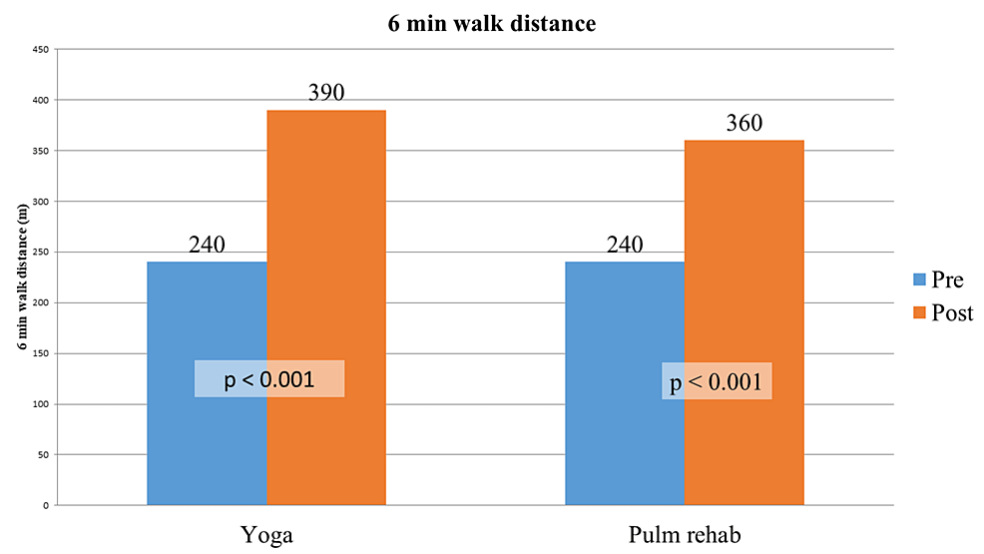
Pre- and post-test of six-minute walk distance

**Figure 3:**
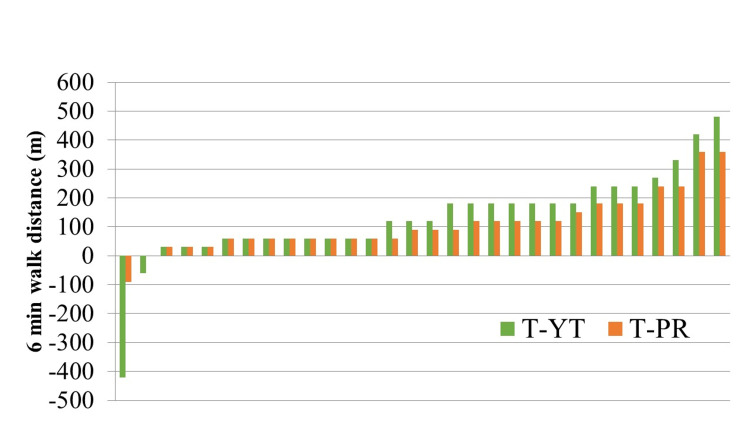
Improvement in six-minute walk distance T-YT - tele-yoga therapy; T-PR - tele-pulmonary rehabilitation

In the third month of 6MWD after T-YT and T-PR, both groups showed improvement. 6MWD for the T-YT arm at baseline, a median was 240 (range: 120-510), and for the T-PR arm at baseline, a median was 240 (range: 120-660). After a three-month follow-up, the median for the T-YT arm was 390 (range: 60-720), and for the T-PR arm, the median was 360 (range: 240 and 630). 6MWD for the T-YT arm was higher. Furthermore, there were no significant differences between the control and intervention groups in terms of 6MWD improvement (p=0.333; Figures 2-3, Table 3).

**Table 3:** Study parameters at baseline and third-month follow-up 6MWD - six-minute walk distance; CAT - COPD assessment test; mMRC- modified medical research council scale; SGRQ - St. George respiratory questionnaire; PHQ-9 - patient health questionnaire; GAD-7 - generalised anxiety disorder scale; FEV1% - forced expiratory volume in one second; COPD - chronic obstructive pulmonary disease; T-YT - tele-yoga therapy; T-PR - tele-pulmonary rehabilitation

Parameter	Baseline		Third-month follow-up	Improvement from baseline for mean±SD/median (range)	P-value (third-month follow-up)
6MWD median (range)	T-YT	240 (120-510)	390 (60-720)	120 (60-240)	<0.001
	T-PR	240 (120-660)	360 (240-630)	90 (60-180)	<0.001
FEV1% mean±SD	T-YT	47.4±13.0	49.2±15.7	1.83±9.05	0.276
	T-PR	52.9±20.4	53.4±19.4	0.47±11.19	0.821
CAT score mean±SD	T-YT	13.7±4.7	7.4±2.6	6.3±5.4	<0.001
	T-PR	14.6±4.8	7.6±3.3	7.0±5.0	<0.001
mMRC median (range)	T-YT	3 (1-4)	3 (2-4)	1 (0-2)	<0.001
	T-PR	2 (0-3)	2 (1-3)	1 (0-2)	<0.001
SGRQ mean±SD	T-YT	50.35±13.70	48.91±48.84	23.0±15.1	<0.001
	T-PR	27.36±11.64	30.64±11.95	18.3±15.2	<0.001
PHQ-9 mean±SD	T-YT	10.6±3.6	4.7±3.0	5.9±3.9	<0.001
	T-PR	9.7±3.9	4.7±2.3	5.1±3.7	<0.001
GAD-7 mean±SD	T-YT	8.7±3.7	6.8±3.6	4.6±3.4	<0.001
	T-PR	4.1±2.1	4.0±2.7	2.7±2.9	<0.001

CAT score and mMRC score

CAT score improved post-intervention in the T-YT arm, as well as in the T-PR arm (p<0.001). There was no significant difference in the improvements in CAT scores between the two groups (p=0.637). Similarly, mMRC scores showed significant improvement in both arms at the end of the third month, the T-YT arm (p<0.001) and T-PR arm (p<0.001), but no significant difference in the improvement of mMRC scores between the T-YT and T-PR arms (p=0.498).

FEV1%

FEV1% showed no statistically significant difference within the two arms at the end of three months (p>0.05) or between the two arms (p=0.605).

SGRQ score

There was a significant difference within the group in T-YT as well as in the T-PR arm (p < 0.001) and no statistically significant difference between the two groups at the end of the intervention (p=0.232).

PHQ-9 and GAD-7 score

A statistically significant difference between the T-YT and T-PR arms within the group (p<0.001) was seen, but the difference between the two groups was not statistically significant (p=0.385). Paired t-test showed that significant differences exist between baseline and third-month GAD-7 scores in the T-YT group (p<0.001) and between baseline and third-month in the T-PR group (p<0.001) but no significant difference in the improvement of GAD-7 score between the two groups (p=0.605; Table 3). Both T-YT and T-PR were safe, and during the course of the trial, there were no reports of any adverse events or periods in either group.

## Discussion

Sixty patients diagnosed with COPD were recruited from a tertiary care center for this study (AIIMS, Rishikesh). Thirty patients were randomly assigned to either the T-YT or T-PR arm.

PR is an integral part of non-pharmacological management in COPD [17]. The COVID-19 pandemic forced a shift from onsite to online interventions, including a shift from institutional PR to tele-PR. Tele-PR is advantageous as it is more convenient and less expensive though it needs an internet network and compares superiorly to home-based PR in terms of supervision [10].

Tele-rehabilitation for chronic respiratory disorders achieves equal outcomes to center-based pulmonary rehabilitation, without any safety concerns, according to a 2021 international comprehensive study. Tele-rehabilitation for individuals with chronic respiratory illness proved to be effective and safe. This evaluation covered both randomized controlled trials and clinical trials of pulmonary tele-rehabilitation involving 1,904 individuals and five tele-rehabilitation models. There was minimal to no difference in six-minute walk distance, quality of life (SGRQ), breathlessness as evaluated by the chronic respiratory questionnaire (CRQ), or dyspnea score among those with COPD. Tele-rehabilitation had a 93% completion rate (95% confidence interval: 90-96%) compared to 70% for in-person rehabilitation and had no adverse effects compared to in-person rehabilitation or no therapy [18].

A single-blinded, multi-center, randomized controlled trial comparing pulmonary tele-rehabilitation (PTR) to traditional pulmonary rehabilitation included 134 individuals. The purpose of this study was to determine whether pulmonary tele-rehabilitation is superior to standard PR in terms of 6MWD and, secondarily, respiratory symptoms, quality of life (QoL), physical activity, lower limb muscle function, and FEV1% in COPD patients. Patients were randomly allocated to receive either 10 weeks of group-based pulmonary tele-rehabilitation (three 60-minute sessions each week) or regular PR (two 90-minute sessions twice a week). 6MWD did not differ across groups after intervention (9.2 meters, 95% CI: -6.6 to 24.9) or at 22-week follow-up (-5.3 meters, 95% CI: -28.9 to 18.9). There were no significant differences across groups; however, pulmonary tele-rehabilitation (n=57) had more participants who completed the study than PR (n=43; χ2 test p<0.01) [19]. This study demonstrated that pulmonary tele-rehabilitation has similar results to conventional PR in patients with COPD. Similar to the above studies, pulmonary tele-rehabilitation led to improvements in 6MWD, CAT, mMRC scores, SGRQ scores, depression, and anxiety scores without a significant improvement in FEV1% in our study. Yoga therapy (YT) has shown beneficial effects in COPD with some concerns regarding various formats and their safety. Our YT module has been validated by 24 yoga experts and has been utilized in previous studies with no adverse events [7].

The appropriateness and acceptability of tele-yoga for heart failure and COPD patients were investigated in a 2015 controlled pilot research. In this controlled, non-randomized trial, tele-yoga was evaluated for eight weeks with an educational control group (information leaflets mailed to participants with one phone call a week). There were 15 participants, seven in the intervention group and eight in the control group. Participants were linked to biweekly, one-hour tele-yoga classes using internet-connected televisions and multipoint videoconferencing. The intervention was deemed acceptable and adequate by the authors; the intervention arm reported enjoying yoga and appreciating the home-based component, and participants reported a high symptom burden and social isolation. Nevertheless, technological challenges prompted some participants to suffer poor video streaming quality. YouTube contains teaching on physical postures, breathing techniques, relaxation, and meditation. This study demonstrates the acceptability and suitability of T-YT, although it was not compared to conventional YT [20].

YT has the potential to improve various aspects of COPD. A comprehensive review and meta-analysis were conducted using Medline/PubMed, Scopus, and CENTRAL databases to investigate the risks and benefits of yoga for patients with COPD (Cochrane Central Register of Controlled Trials). COPD patients were eligible if they had participated in a randomized controlled experiment assessing the effects of yoga on QoL, dyspnea, exercise capacity, and pulmonary function (FEV1%). The term "safety" refers to an incidental result. Included were the results of eleven randomized controlled studies involving 586 people. Yoga was observed to enhance QoL on the CAT (mean differences [MD]=3.81; 95% CI: 0.97 to 6.65; p=0.009), exercise capacity on the 6MWD (MD=25.53 m; 95% CI: 12.16 m to 38.53 m; p=0.001, I^2^=0%), and lung function on the FEV1% predicted test when compared to no treatment. Only yoga interventions that focused largely on breathing were effective, while those that also involved yoga postures were not. Few instances of negative consequences were recorded. They revealed that yoga offers considerable benefits for COPD patients' exercise capacity and lung function. Similar to the preceding findings, YT benefited in our study in terms of 6MWD and QoL in terms of SGRQ. It was determined to be safe, with no adverse occurrences documented. Unlike the last review or module, this one included breathing, asanas, and kriyas [21].

Using PubMed, Embase, Cumulative Index to Nursing and Allied Health Literature (CINAHL), the Cochrane Library and Web of Science, a second systematic review and meta-analysis on the efficacy of yoga training in COPD patients, identified 10 papers meeting inclusion criteria. Participants in the trial got yoga training, whereas the control group received standard treatment for COPD. 6MWD, Borg scale, estimated FEV1%, SGRQ, and CAT scores were among the outcomes. Yoga teaching resulted in significant improvements in patients' 6MWD, Borg scale scores, FEV1%, partial pressure of carbon dioxide (PaCO_2_), SGRQ, and CAT (p=0.05). The FEV1% and other spirometric measures indicated no statistically significant differences. Similar to this meta-analysis, T-YT in our study did not result in a substantial improvement in FEV1%; however, 6MWD, CAT, mMRC, and SGRQ scores improved significantly [22].

The systematic review and meta-analysis of randomized controlled trials on the effect of PR in COPD patients include studies in which COPD patients engaged in PR regimens based on yoga, tai chi, and traditional physical exercises such as walking, jogging, swimming, and cycling. At least two groups participated in the studies, with one group receiving PR and the other receiving standard treatment. All 39 trials, including 2,397 people with COPD, were included. Compared to those who received conventional treatment, patients who participated in the pulmonary rehabilitation program showed significant improvements in the 6MWD, SGRQ score, and mMRC. Similar to our study, yoga improved 6MWD without affecting FEV1% levels. Similar to the PR group, the T-PR group exhibited enhancements in 6MWD, SGRQ, and mMRC [23].

There are no substantial, well-designed studies comparing the two methods directly. In a meta-analysis, the effects of active mind-body movement therapies (AMBMT) against PR or AMBMT added to PR versus PR alone were compared in individuals with stable COPD. AMBMT includes yoga, tai chi, qigong. Low-quality evidence supports superior disease-specific QoL with AMBMT than PR in stable COPD patients. Poor-quality evidence suggests that there are no differences in dyspnea between AMBMT and PR. Evidence of moderate quality indicates that the addition of AMBMT to PR does not improve disease-specific quality of life. Evidence of lower quality demonstrates that both yoga therapy and pulmonary rehabilitation are safe, effective, and efficient strategies to treat COPD and that both can be utilized alone or as part of an integrated approach by combining their practices for the highest quality results [24].

The integrated approach of yoga therapy (IAYT) helps coal miners with COPD by reducing dyspnea, fatigue, and pulmonary recurrence and enhancing functional performance and peripheral capillary saturation (SpO_2_%). In this randomized, waitlist-controlled, single-blind clinical study, 81 coal miners (aged 36 to 60) with stable stages II and III COPD were included. The yoga group received a 12-week, six-day-a-week, 90-minute-per-day IAYT module for COPD that comprised asanas, loosening movements, breathing practices, pranayama, cyclical meditation, yogic counseling, and lectures. After the intervention phase and post-testing were completed, participants on the waiting list were offered the 12-week yoga program. The yoga group demonstrated statistically significant enhancements in dyspnea (p=0.001), fatigue (p=0.001), pulmonary function (p=0.001), SpO_2_ percent (p=0.001), and 6MWD (p=0.001) [25]. We found similar improvements in symptom scores and 6MWD but not in FEV1%. Our study is an attempt to compare a validated T-YT module with an already proven beneficial T-PR module in patients with COPD. It shows that a well-designed T-YT module can benefit similar to the T-PR module in terms of exercise capacity, symptom burden improvement, QoL, and psychiatric co-morbidities like anxiety and depression, which are common [26].

The progressive nature of COPD, distance issues, and the COVID-19 pandemic have put the onus on the ever-expanding role of tele-interventions in these patients. As mobile technology increases its reach to far-flung areas and internet connectivity improves, the role of these tele-interventions is destined to expand.

Limitations

Our research is restricted in various ways due to the fact that it is a pilot study conducted at a single location with a small sample size.

## Conclusions

Our preliminary pilot study using a validated T-YT module demonstrates that a three-month T-YT is comparable to a T-PR in terms of exercise capacity, symptom scores, SGRQ, PHQ-9, and GAD-7. Additional research with a larger sample size is necessary to verify these findings. However, because T-YT is simple, inexpensive, and risk-free, it has the potential to be adopted as part of non-pharmacological therapy in COPD patients.
